# Application of ultrasound to monitor in vivo residual bone movement within transtibial prosthetic sockets

**DOI:** 10.1038/s41598-024-60353-7

**Published:** 2024-04-27

**Authors:** Niels Jonkergouw, Maarten R. Prins, Daniël Donse, Peter van der Wurff, Jaap H. van Dieën, Arjan Buis, Han Houdijk

**Affiliations:** 1 Department of Orthopaedic Technology, Military Rehabilitation Centre Aardenburg, Korte Molenweg 3, 3941 PW Doorn, The Netherlands; 2 Department of Human Movement Sciences, University Medical Centre Groningen, Groningen, The Netherlands; 3 Department of Human Movement Sciences, Vrije Universiteit Amsterdam, Amsterdam, The Netherlands; 4 Department of Mechanical Engineering, TU Delft, Delft, The Netherlands; 5 Department of Biomedical Engineering, Strathclyde University, Scotland, UK

**Keywords:** Rehabilitation, Bone imaging, Biomedical engineering

## Abstract

Transtibial prosthetic users do often struggle to achieve an optimal prosthetic fit, leading to residual limb pain and stump-socket instability. Prosthetists face challenges in objectively assessing the impact of prosthetic adjustments on residual limb loading. Understanding the mechanical behaviour of the pseudo-joint formed by the residual bone and prosthesis may facilitate prosthetic adjustments and achieving optimal fit. This study aimed to assess the feasibility of using B-mode ultrasound to monitor in vivo residual bone movement within a transtibial prosthetic socket during different stepping tasks. Five transtibial prosthesis users participated, and ultrasound images were captured using a Samsung HM70A system during five dynamic conditions. Bone movement relative to the socket was quantified by tracking the bone contour using Adobe After-Effect. During the study a methodological adjustment was made to improve data quality, and the first two participants were excluded from analysis. The remaining three participants exhibited consistent range of motion, with a signal to noise ratio ranging from 1.12 to 2.59. Medial–lateral and anterior–posterior absolute range of motion varied between 0.03 to 0.88 cm and 0.14 to 0.87 cm, respectively. This study demonstrated that it is feasible to use B-mode ultrasound to monitor in vivo residual bone movement inside an intact prosthetic socket during stepping tasks.

## Introduction

Transtibial prosthetic users commonly encounter difficulties in achieving an optimal fit between their residual limb and socket, with a reported prevalence of 59% experiencing moderate to severe residual limb pain^1,2^. Simultaneously, prosthetists encounter difficulties in objectively evaluating the effects of their prosthetic adjustments on the loading of the residual limb^3,4^. Currently, prosthetic fitting relies on iterative processes, employing a trial-and-error approach to improve residual bone control. However, when utilizing a socket suspended prosthesis there is no immediate control over the skeletal system. Consequently, the residual bone may move in relation to the socket, leading to soft tissue deformations within the residual limb during prosthetic use. These deformations of stump tissues will cause stress within these tissues^5^, potentially resulting in discomfort, pain, and tissue breakdown. Over time, these issues could lead to disuse and reduced functioning^2,5^.

Clinicians focus on enhancing the stability of the residual limb in the socket, reducing the residual bone movement with respect to the socket, to influence soft tissue deformations. However, there is a lack of comprehensive understanding on the mechanical behaviour of this connection. Characterizing the mechanical behaviour of the residual limb movements would help to comprehend how adjustments to the prosthesis may impact the stability of the residual limb within the socket, as well as its effects on tissue deformations and resulting stress and strain^6^. Visualization of internal bone movement within the prosthetic socket holds the potential to provide the insights needed to better understand the dynamics of interactions between the residual bone and the socket^7,8^.

To quantify and understand the mechanical interactions within the socket, measurements can be conducted with stress and strain sensors at the stump-socket interface^9,10^. These insights are considered useful in guiding a prosthetist in making informed decisions by assessing alterations in interface stress following prosthetic interventions^10^ and can be used as an indicator of the quality and comfort of the fit^11^. However, while these interface pressure measurements may explain the reactive forces affecting the superficial skin level, they fall short of providing a comprehensive understanding on the actual stress and strain within the stump tissues^7^. Stress and strain measurements on the socket interface will not provide insight in the deeper tissues deformations by residual bone movement^5,12,13^, nor in the stability of the connection between residual bone and socket.

Conventional imaging techniques, such as X-rays^14,15^, have been used to investigate sagittal plane movement of the bone within the socket. Lilja et al.^15^ and Grevsten et al.^14^ investigated gait by obtaining four images representing different gait phases. They respectively reported a mean 2.2 cm and a 4.5 cm anterior–posterior movement of the distal part of the tibia bone within the socket. Their technique offered high contrast visualization of the residual bone. However, these radiological images are captured in static situations to represent a specific gait phase and thus miss any dynamic effects. Additionally, they cause harmful radiation exposure. Alternatives like CT^16^ scans and MRI come with accessibility and costs issues, in addition to the limitation of capturing a static gait phase representation^17^, making such systems mainly suitable for volumetric assessments and differentiation of tissue layers in static situations^18^. Thereby, frontal plane’s residual bone movement have not been investigated in those studies. This highlights the challenge of implementing advanced diagnostic systems to measure residual bone movement during dynamic tasks^7,8,19^.

Ultrasound (US) has been employed for decades to monitor various musculoskeletal dysfunctions^20^. This relatively cheap and simple method could potentially be utilized as a diagnostic or investigational tool for understanding the residual bone-socket interactions and deformation of soft tissues by prosthesis users^3,7,8^. However, the application on prosthetic devices is limited. Most US studies in the field of prosthetics present the conceptual design of a measurement system, without actually measuring during prosthesis use^17,18,21–23^. The first dynamic measurements have been reported in two studies on a single transfemoral prosthesis user during daily life activities and prosthetic gait^24,25^. The method used, however, involved creating large cavities in the socket to gain access to the skin for the US transducer^23–25^. Such socket adjustment would influence the tissues of the limb and consequently affect deformations of the soft tissues in the area of interest^18^.

Utilizing US to measure residual limb movement in transtibial amputees presents a viable approach for several reasons. Unlike other imaging and assessment methods, US offers real-time insights into dynamic movement, enabling a more comprehensive understanding of residual bone-socket interactions and soft tissue deformation during prosthesis use. By capturing the movement in both sagittal and frontal planes, US has the potential to provide dynamic, non-invasive, and non-ionising measurements of relative movement between bone and socket^7^. The aim of this study was to assess the feasibility of a new approach to apply B-mode US to monitor the in vivo movement of residual bone within the socket during stepping tasks. To this end, we first describe the development of a method to apply B-mode ultrasound, subsequently we assess precision of the outcome in terms of signal-to-noise ratio and finally we describe the magnitude of bone movement during stepping tasks.

## Methods

### Participants

This study comprised a convenience sample of five participant selected consecutively following a call for participants. They were included between February-June 2022 and were considered eligible to participate if they were at least 18 years old, had used a transtibial prosthesis for a minimum of 1 year, were able to walk without additional walking aids, and had a stump which was deemed suitable for sub-atmospheric pressure fitting by a certified prosthetist. Participants were not eligible when they presented themselves with stump problems, cognitive or communicative disorders or visual impairments which would limit their balance.

This study was approved by the Medical Ethical Review Board of the University Medical Centre Groningen, Groningen, the Netherlands (NL74038.042.21). All participants provided written informed consent prior to the start of the study. This quantitative, case-series study was conducted according to the principles of the Declaration of Helsinki^26^.

### Equipment

#### Ultrasound apparatus

A Samsung HM70A US system (Samsung Healthcare, the Netherlands) was used to acquire US images in the transverse plane of the residual limb. The B-mode US images were captured with a linear array transducer (LA3-16AD, with a bandwidth of 3.0–16.0 MHz, field of view: 38 mm). All measurements were executed at the Department of Human Movement Sciences, VU Amsterdam, Amsterdam the Netherlands.

The HM70A system was set to the following settings: 8.8 MHz/Frq Pen. 4.5 cm, Frame Avg 8, Dynamic range 125, Reject level 1, Gray Map 13, MultiVision Middle, ClearVision Off, Scan Area 100%, Tissue 1500 m/s, Edge Enhance 0, Focus Number 1, Power 100% and a FSI of 1.

### Protocol

**Figure 1:**
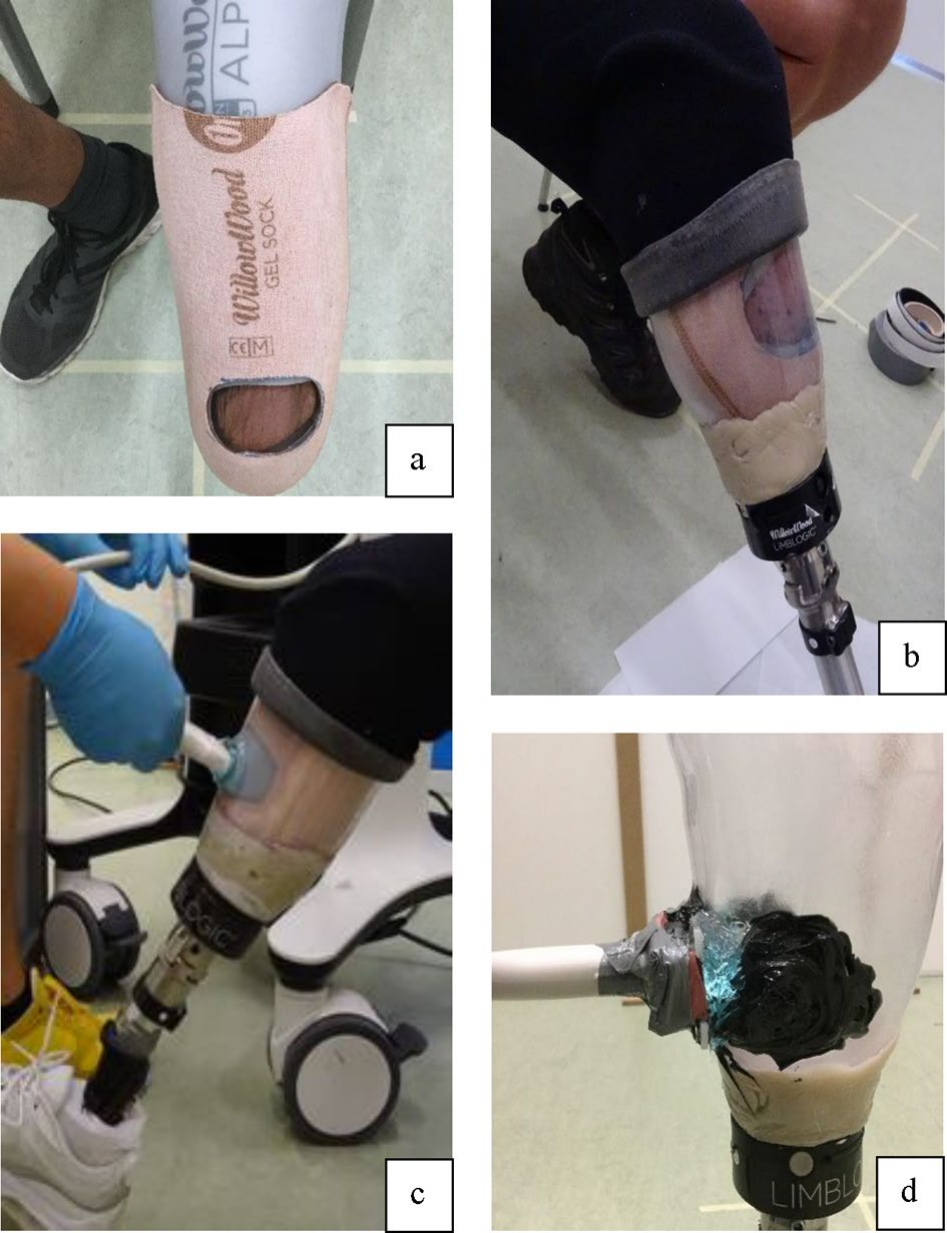
Process of probe placement. (**a**) The silicone-gel liner is the grey liner, worn directly on the skin. A brown, gel-sock has been donned over the liner. Two holes have been cut, one in the liner and one in the gel-sock. As a result, the skin is shown at the area of interest. Ultrasound gel was used to fill the area of the removed material. (**b**) The socket was donned with the black vacuum sleeve enabling a vacuum within the socket. The gel was used as a medium to fill the cut-out material of liner and gel-sock. The active vacuum fitting prevented the gel to move away from the area of interest. (**c**) Ultrasound gel was applied to the external part of the socket. Followed by the determination of the correct position for the US probe. This was deemed successful when a clear and stable image of the anterior margin of the tibial bone could be captured. Once this position was identified, the probe was securely fixed to the socket using a custom-made clamp. (**d**) When the probe was connected with the clamp the remaining gaps were filled with ultrasound gel. Thereafter everything was covered with duct tape to hold the ultrasound gel in place.

Upon enrolment in the study, all five participants underwent a standard prosthetic fitting procedure to acquire a total surface bearing sub-atmospheric pressure socket. They received a gel DUO-liner, existing of a Silicon-gel liner (Willow Wood Global, Ohio, USA) and a gel-sock (brown sock, Fig. 1, top left), a Thermolyn Clear socket (616T83 = 10, Otto Bock, Duderstadt, Germany), a Derma Proflex vacuum sleeve (435A3, Otto Bock, Duderstadt, Germany) and a distal mounted Limblogic active vacuum pump (LLV-2000-L, Willow Wood Global, Ohio, USA). All participants used their own prosthetic foot during the experiments. The process of fitting and alignment was comparable to clinical care, with socket rectifications on the locations where the stump/socket connection needed to be improved based on subjective observations of patient and certified prosthetist. As soon as the prosthetic fit was judged sufficient, the research measurement appointment was made.

#### Ultrasound probe positioning

US gel was applied to the distal portion of the tibial anterior crest before the participant donned their liner. Hereafter, a gel-sock was donned (brown sock in Fig. 1a) in which a gap was created at the area of interest to minimize the material through which the US waves needed to travel. A clear contrast of the residual bone relative to the surrounding tissue was detected through the socket when positioning the probe. However, after participant 1 and 2, a design change was implemented. This adjustment was deemed necessary since the residual bone movement was repeatedly lost from the US images during the stepping tasks of participant 1 and 2. Therefore, an additional gap was created in the liner for the consecutive participants (Fig. 1a).

The residual tibial movement with respect to the socket was expected to be most prominent at the distal part of the residual bone. Therefore, the gap was created as much distal as possible, while still allowing a perpendicular orientation of the probe relative to the residual bone. Hereafter, US gel was applied in the gap of the liner and gel sock to compensate for the lost material and to prevent air to creep inside the socket. Subsequently, the participant donned their prosthesis, rolled on the suction sleeve, and activated the vacuum pump in an upright standing position. Sub-atmospheric pressure fitting was achieved, preventing air to influence the US measurements. Sub-atmospheric pressure fitting was deemed necessary for this study, as in a pilot-test on a prosthetic user with a pinlock system, the residual bone moved away from the socket, allowing air to enter the resulting gap, causing a black image.

After preparing and donning the prosthesis, the participant returned to a seated position whereafter the ultrasound probe was rigidly connected to the outside of the socket covering the location where a gap in the liner was created (Fig. 1b). The correct probe position was determined by visually optimizing the contrast between the socket, liner, skin, and residual bone within the ultrasound image. The probe was rigidly fixed, perpendicular to the stump, in a custom-made 3D-printed clamp (Ultimaker 3, Utrecht, the Netherlands) that was glued (+PLUSeries 60 Second Adhesive, Fabtec, Everett, Washington, USA) to the socket. Gel was inserted in the cavity between the clamp and the socket wall to fill the remaining gaps and prevent air between probe and socket. Duct tape was applied around the clamp to prevent the US gel to run out from under the probe during the measurements.

### Procedure

**Figure 2:**
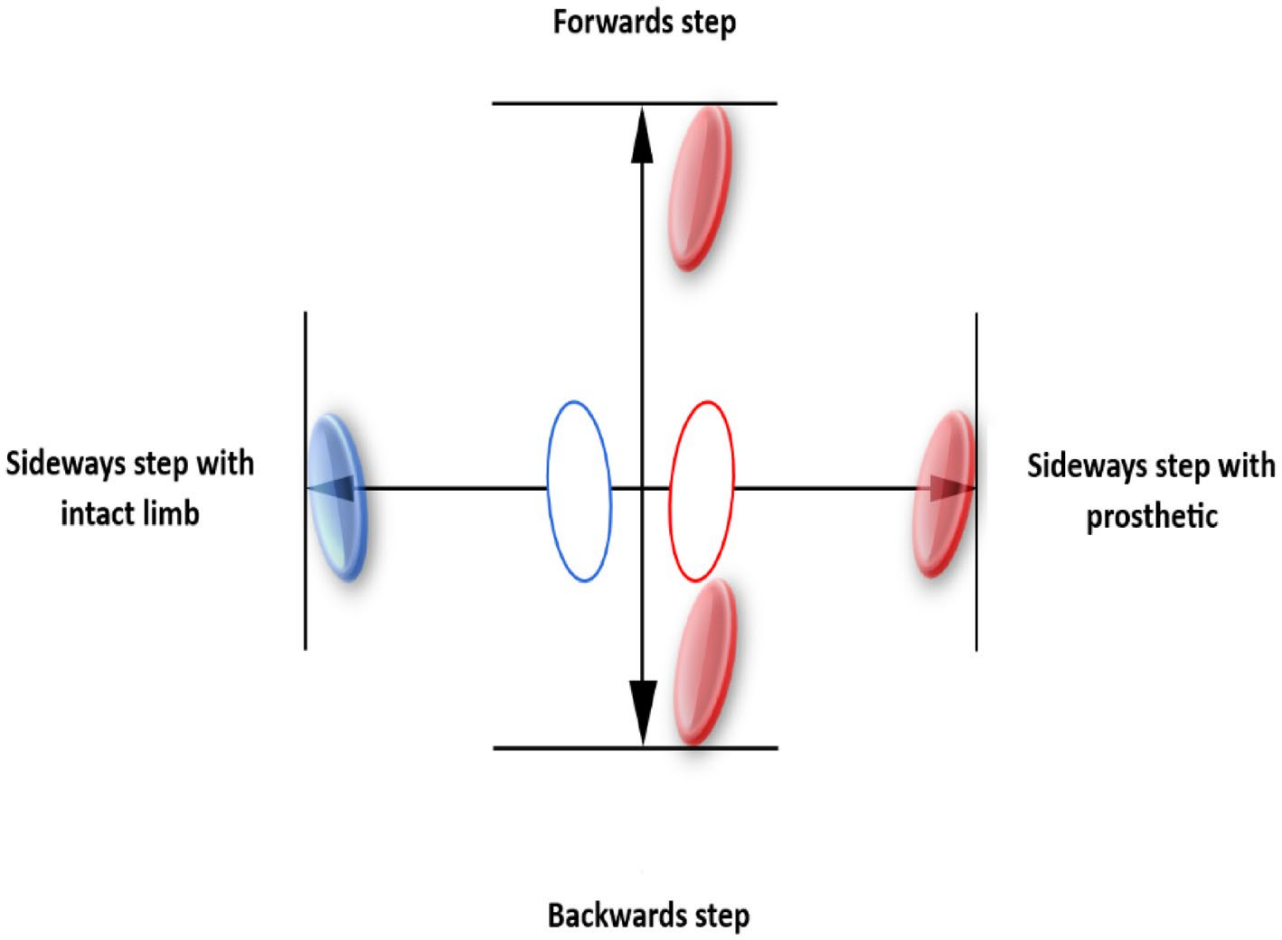
Setup of the stepping task. The stepping task was initiated from the starting position in the middle of the cross where two open ellipses represent the feet, intact limb (blue) and prosthetic limb (red). After each step the participant returned to the starting position. For each condition the step distance was determined as illustrated by the line at the end of the arrows. For forward and sideways, this distance was set at 33% of the height of the subject and indicated with a line on the floor. For backwards steps, this was half the distance of the other steps. The filled ellipses represent the approximate target locations of the foot.

Each participant was requested to stand in an upright position at the centre of a cross, which was outlined on a flat ground surface. The dimensions of the marked cross were determined based on the participant's height and were indicated at each end of the cross on the ground. For forward and sideways steps, the distance was set at 33% of the participant's height, while the distance for backward steps was half that of the other steps (Fig. 2). We have arbitrarily selected these distances base on the height of our participants so that the step sizes were achievable and comfortable for all participants.

The stepping trials were started with ten weight shifts between prosthetic and intact leg, the “in place” condition. In bipedal stance, the body weight was shifted onto the prosthesis until the intact limb was only touching the ground for balance. After each weight shift the participant returned to a stable stance. Next, ten “forward” steps were conducted with the prosthetic limb, followed by ten “backwards” steps. Finally, the participant executed the sideways steps, starting with ten steps with the “prosthetic limb”, followed by ten steps with the “intact limb” (Fig. 2).

During all five conditions the participant was instructed to put body weight on the prosthesis until their weight was almost completely supported by the prosthetic leg, with only the toes of the intact limb touching the floor. Throughout the measurements, a metronome set at 50 bpm was utilized to guide the movement speed during the trial. Cycles started on the first beat, taking body weight on the stepping leg second on the second, starting the return to baseline on the third, and returning to the baseline position on the fourth beat.

### Data recording and analysis

The participants’ movement in the frontal and sagittal planes was recorded on digital video using an iPhone 8 capturing 1080p at 60fps and an iPad 2 capturing 720p at 30fps (Apple, Cupertino, California, USA) positioned at 2 m distance from the participant. Both video recordings were started while capturing the screen of the Samsung HM70A US system, such that the time code of the US recordings could be seen. Thereafter, the US video was started which would later serve as a synchronization point between the videos and US video.

All videos were synchronized using Premier Pro software (Adobe, San Jose, California, USA) Subsequently, weight shift/step initiation and termination were determined from visual analysis of the frontal and sagittal videos. The start of body shifting or sideward movement to the non-prosthetic side was used as criteria for the “in place” and “intact limb” conditions respectively. With the other steps it was considered to begin when progression of the prosthetic socket was detected on the prosthesis. For all five conditions the end was determined as the instant the participants came to a complete stop in the baseline position. The stop of external motion of the socket/participant may not precisely align with the internal bone movement. Therefore, an additional five US frames were included at the beginning and end of each step.

The first and last step were omitted from the analysis to take into account deviations caused by starting and stopping within the cyclic stepping tasks. The start and end timings for the remaining eight steps per condition were exported and used to segment the US data.

The US movie (.mpg) was imported in After Effects (Adobe, San Jose, California, USA). This allowed us to attach a semi-automated tracker on the most prominent point of the tibial crest and write the x (anterior–posterior) and y (mediolateral)coordinates of the trackers movement to the null-layer. The confidence level of this tracker was set to 80%, when dropping below that confidence level, the video was paused, and manual correction of the marker was performed if needed. Once the tracking process was completed, the x and y image coordinates of the null layer, along with the corresponding time stamps, were exported in a .csv format.

### Analysis

The precision of bone movement tracked from the US data was determined through the calculation of the signal-to-noise ratio (SNR). This SNR quantified the ratio of the average bone movement to the standard deviation of movements over repetitions, representing combined movement variability and measurement noise. Initially, each individual time series was normalized to 101 samples per step execution. These 101 samples per step were demeaned, in other words, the average x and y position was set to zero for each step. Subsequently, the mean movement trajectory over the series of steps (consisting of 101 data points each) was determined, resulting in 30 mean movement trajectories (3 participants × 5 conditions × 2 planes). For each of these trajectories, the root-mean-squared value (‘signal strength’) was calculated. Subsequently, the ‘noise strength,’ the root-mean-squared-difference of the individual repetitions relative to the mean trajectory was calculated. Finally, the former was divided by the latter, resulting in the SNR.

The absolute range of motion of the bone relative to the socket was assessed as the absolute difference between the minimum and maximum values of each mean trajectory. Data analyses and data visualization were conducted using MATLAB 2018A (The MathWorks, Inc., Natick, MA).

## Results

### Demographics

All participants were male, with ages ranging from 47 to 68 years, weights between 72 and 110 kg, heights between 1.74 and 1.93 m, and stump lengths between 0.11 and 0.19 m. The time since amputation for the participants varied from 6 to 51 years. Four had their amputation due to trauma and one as a result of an infection with Clostridium Myonecrosis. All participants used an Elastic Response Foot and were classified with a high activity K-level 4 (Table 1).

**Table 1:** Group demographics.

#	Age (y)	Weight (kg)	Length (cm)	Time since amputation (y)	Cause of amputation	Side of amputation	Effective stump length (cm)*	Socket length (cm)**	Probe placement (cm)***	Prosthetic foot (company)	ADL prosthesis fitting
1	68	90	180	51.0	Trauma	Right	14.0	18.2	7.5	Triton (Otto Bock)	Pin fixation
2	51	72	174	18.0	Trauma	Right	12.0	15.5	8.7	Triton (Otto Bock)	Pin fixation
3	51	84	182	6.0	Trauma	Left	19.1	16.8	10.2	Triton VS (Otto Bock)	Pin fixation
4	50	110	192	34.0	Infection	Right	17.0	17.9	12.0	Triton (Otto Bock)	Conventional with thigh corset and hinge
5	47	86	193	17.0	Trauma	Right	11.2	11.8	8.6	All-Pro (Fillauer)	Active vacuum

Group’s mean value ± standard deviation. The two participants highlighted with grey were excluded from the analysis in relation to the quality of the video of the residual limb movement.

*ADL* Activities of Daily Living, *cm* centimetres, *kg* kilograms, *SD* standard deviation, *y* years

*The effective stump length is measured by taking the perpendicular distance of the medial tibia plateau to the most distal part of the residual tibia bone.

**Socket length is the most distal internal part of the socket up to the medial tibia plateau.

***Probe placement is the length of the middle of the probe to the medial tibia plateau.

### Ultrasound image quality

**Figure 3:**
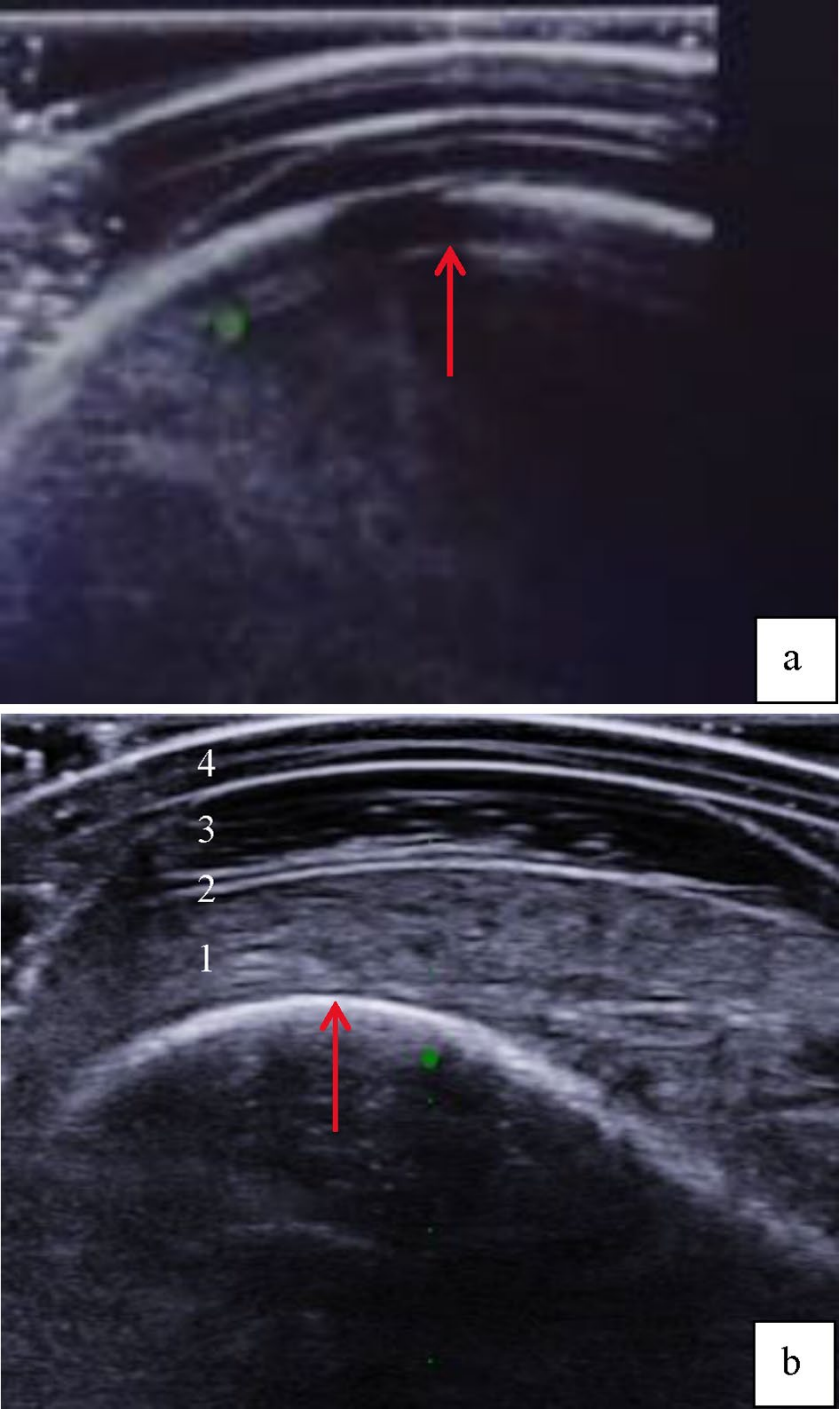
Screen capture of the ultrasound measurements comparing the contrast of the residual bone for participant 1 (**a**) and participant 3 (**b**). The two images displayed illustrate the variations in bone contrast, as indicated by the red arrow, within the ultrasound image. While comparing the two images, it becomes evident that the clarity and contrast in the bottom image (representing participant 3, 4 and 5) far surpasses the quality of that seen in the top image. Within the lower image, a clear distinction between the various layers is depicted. The red arrow points to the bone, while the grainy structure represents the (1) soft tissue over the tibia. Followed by a distinct line denoting the (2) superficial skin. Thereafter, the blackish area indicates the presence of (3) ultrasound gel, which fills the space under the (4) prosthetic socket, shown by two sharp lines, visible at the top of the image.

The edge of the bone within the US data of participants 1 and 2 proved to be insufficiently visible for accurate monitoring during stepping (Fig. 3). Therefore, their data were omitted from the analysis. The described protocol adjustment (*Methodology: Ultrasound probe positioning*) at the area of interest resulted in a clear contrast of the residual bone with respect to tissue and socket layers for participants 3, 4 and 5. An example is presented in Fig. 3.

### Bone range of motion

**Figure 4:**
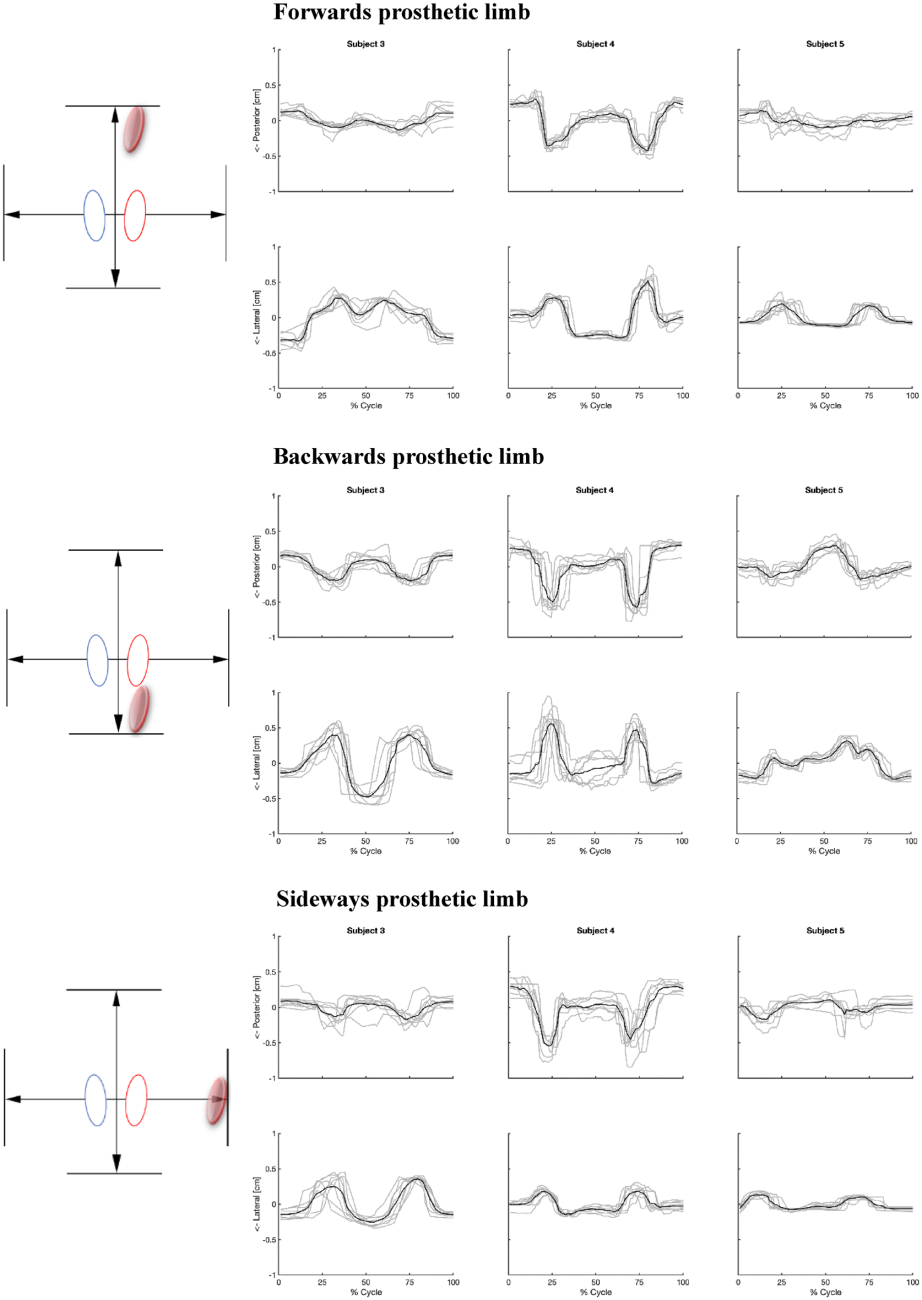
Measured residual limb movement with prosthetic steps. Each grey line corresponds to the residual bone movement during a single step in the prosthetic step conditions. The mean trajectory is depicted by the black line. The zero axis is positioned at the mean of the trajectory, which was utilized for the signal-to-noise ratio, representing the data most accurately. The top three images illustrate the anterior/posterior motion, while the bottom three depict the medial/lateral motion of the residual bone. To note: the four metronome beats correspond approximately to the 0% mark (step initiation from baseline), 33% mark (step landing), 67% mark (returning step initiation), and 100% mark (baseline position).

**Figure 5:**
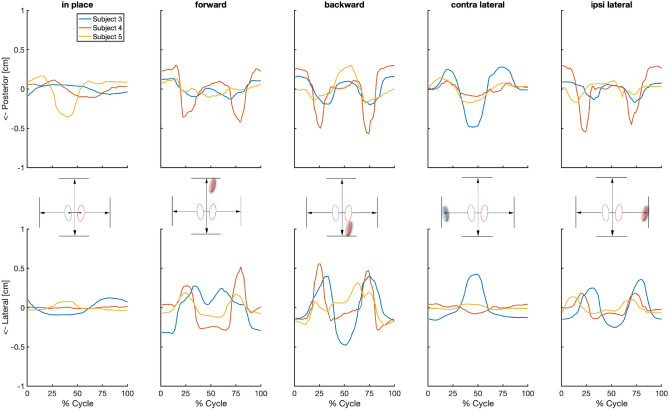
Mean values of absolute residual bone movement for each trial, compared among participants within the same condition. The y-axis represents anterior–posterior and medial–lateral motion within the prosthetic socket. The direction of motion is determined from the starting point of each executed step and is presented as the absolute movement from that specific point as a percentage of the executed step (x-axis). To note: the four metronome beats correspond approximately to the 0% mark (step initiation from baseline), 33% mark (step landing), 67% mark (returning step initiation), and 100% mark (baseline position).

The bone movements during the repetitive stepping tasks for all three participants and conditions are presented in Fig. 4. Consistent bone movement patterns were observed for all participants and all stepping directions (Supplementary Video S1, a representation of this bone movement). The SNR values over all participants and conditions (Table 2) ranged from 1.12 to 2.33 (Table 2). The range of motion of the mean trajectory in the medial–lateral and anterior–posterior directions ranged from 0.03 to 0.88 cm and from 0.14 to 0.87 cm respectively, with considerable differences between participants within the same conditions (Table 2, Figs. 4, 5, Supplementary Figs. S2, S3).

**Table 2:** Absolute bone movement.

Participant number	Medial–lateral				Anterior–Posterior			
	Mean	Signal strength	Noise strength	SNR	Mean	Signal strength	Noise strength	SNR
**Forwards prosthetic step**								
3	0.61	0.17	0.07	2.64	0.26	0.07	0.05	1.35
4	0.80	0.18	0.06	3.16	0.72	0.18	0.06	2.90
5	0.32	0.09	0.04	1.98	0.25	0.05	0.06	0.79
Mean SNR		0.15	0.06	2.59		0.10	0.06	1.68
**Backwards prosthetic step**								
3	0.88	0.23	0.11	2.12	0.36	0.12	0.06	2.03
4	0.84	0.18	0.15	1.24	0.87	0.20	0.10	1.92
5	0.54	0.13	0.05	2.49	0.48	0.11	0.07	1.56
Mean SNR		0.18	0.10	1.95		0.14	0.08	1.84
**Sideways: prosthetic limb**								
3	0.61	0.17	0.09	1.93	0.26	0.07	0.08	0.91
4	0.33	0.08	0.05	1.62	0.84	0.18	0.12	1.55
5	0.21	0.06	0.03	1.94	0.28	0.06	0.06	0.91
Mean SNR		0.10	0.06	1.83		0.10	0.09	1.12
**Sideways: intact limb**								
3	0.58	0.15	0.04	3.58	0.76	0.19	0.08	2.48
4	0.13	0.03	0.02	1.31	0.20	0.05	0.05	0.88
5	0.07	0.02	0.01	2.09	0.32	0.08	0.04	1.93
Mean SNR		0.07	0.02	2.33		0.11	0.06	1.76
**In place**								
3	0.22	0.08	0.03	2.93	0.14	0.04	0.04	1.10
4	0.03	0.01	0.01	0.51	0.22	0.06	0.03	1.77
5	0.11	0.03	0.02	1.40	0.52	0.14	0.08	1.60
Mean SNR		0.04	0.02	1.61		0.08	0.05	1.49

The mean range of motion is calculated by determining the absolute difference between the highest and lowest outcomes within the mean trajectory of each condition. All measurements are converted to centimetres and recorded in terms of mean with standard deviation over eight steps. The root-mean-square error of the amplitude is the signals strength, and the root-mean-square-difference is representing the signals noise. Hereafter, a signal-to-noise ratio (SNR) is calculated by dividing the root-mean-square amplitude by the root-mean-square-difference. For each condition a mean SNR was calculated for medial–lateral and anterior–posterior direction.

## Discussion

We present an US method to obtain clear contrast of the residual bone through a prosthetic socket, to track the residual bone movement during dynamic tasks, without affecting the integrity of the prosthesis. The SNR values suggests reasonable precision in utilizing US across various conditions and participants. The observed movement pattern showcases consistency of residual limb movement, alongside individual differences of amplitudes, which could reach up to 0.87 cm within anterior–posterior as well as medial–lateral direction.

The experimental conditions were characterized by repetitive bone movements, synchronized with the frequency of the steps performed. SNR values indicate amplitudes greater than the measured movement variability and measurement noise. The within-subject noise may result from measurement errors, as well as variations in step execution. Between participant variations could as be influenced by, stump circumference, soft tissue properties, prosthetic alignment, components, measurement location, and socket fit. Even though the exact source of the variation cannot be pinpointed, the SNR values demonstrate that the mean bone movement can be picked up from the potential noise in the ultrasound images across different participants and conditions, even with a limited number of steps (n = 8). This supports the feasibility of the proposed method. The SNR does not only contain measurement noise but also common step variability, which adds to the SNR value. Nevertheless, capturing the variance across repeated steps could prove to be valuable in prosthetic fitting research. Variation, particularly the extreme values in the distribution, might be closely linked to stump tissue damage.

The three prosthetic stepping conditions: “Forwards”, “Backwards” and “Side-ways,” demonstrated a consistent bi-phasic pattern of motion in the medial–lateral as well as the anterior–posterior direction. The observed similarities between these prosthetic steps conditions suggest that, when executing the prosthetic step, there is a consistent residual bone movement. These patterns can be accounted for in the light of the timing of the imposed movement with the beats of the metronome. In each condition the starting point was from an upright standing position on both legs. Maximal posterior residual bone movement looks to be present within the swing phases of the executed step, approximately between 0–33% (1st to 2nd beat) and 67–100% (3rd to 4th beat) of the normalized cycle time. This movement is likely caused by swinging the prosthesis forward, after which the inertial forces on the prosthesis would lead to the residual bone moving away in respect to the socket wall or vice-versa. Preceding the swing phase the residual bone movement became more anterior orientated, probably due to applying weight on the prosthesis (33–67%, 2nd to 3rd beat). Compared to the aforementioned conditions, a different motion pattern was observed in the: ‘Intact Limb Sideward step’ and ‘weight shift In Place’ conditions. Given that these conditions do not involve a swing phase of the prosthetic limb, the described range of motions differs. In the ‘Sideways: Intact Limb’ condition, a different peak is observed when stepping toward the intact limb. As the executed motion is distinctively different it suggests that in this case the balancing function of the prosthetic leg results in different residual bone movements. These movement patterns and directions observed within this study can be explained by the mechanical reasoning of expected bone movements during the executed stepping task. While more elaborate tests of validity need to be performed, these results indicate that information on the validity of the measurements is present in the current study design, particularly in terms of face validity.

The mechanics of the connection between the socket and the stump are not well-defined^2^. US measurements could show movement of the residual bone with respect to the socket and may be of use in understanding the relationship of residual bone movement to internal stress and strain of the soft tissue layers. Some studies have tried to understand the residual limb movement by investigating the socket-stump interface with surface measurements^7,27^. Our study visualizes the residual limb movement with respect to the socket and indicates that studies should consider this stump-socket connection as a pseudo-joint with its own characteristics and not as a stiff connection between skeletal system and prosthesis. It would be interesting to investigate the combination of measurement of stress, strain and of residual bone movements. This could be used as input to modelling studies to investigate the effect of socket design changes on residual bone movement and tissue damage^7,28^.

Within this study the probe was placed at the most distal part of the residual bone, which was not the same relative distance from the knee between participants, because residual bone lengths varied. We chose this approach because residual movement at this location was expected to be largest. However, it's important to note that the extent of residual bone movement may vary depending on the length of the residual bone, which could contribute to the variation observed between participants. Additionally, the US videos were obtained in the transverse plane and therefore, longitudinal displacement may have influenced the results as well. Future research can address these challenges by aligning two probes at different levels along the longitudinal axis of the stump. Integrating data from these probes would enable the calculation of the axis of motion of the residual bone, moving beyond the single transverse plane assessment of socket motion, as demonstrated by Convery et al. in a transfemoral amputee study^24^. Furthermore, combining this data with ground reaction forces would provide additional insights into the mechanics of stump-socket interactions during prosthetic use and is strongly recommended.

A limitation of this feasibility study is the inclusion of a small sample of the targeted population. However, the main aim of this study was to test the feasibility of using B-mode ultrasound to monitor in vivo residual bone movement within a transtibial prosthetic socket. This pilot study demonstrated the possibility to detect in socket bone movement during stepping tasks and showed its magnitude and variation amongst participants. This simulates the need for future research to include a larger sample size to thoroughly evaluate bone movements within the studied population and to conduct further validity testing on both the introduced design and the settings of the ultrasound system used during the measurements. While ultrasound holds promise for understanding residual limb movement within clinical settings, its direct implementation is currently constrained by its cumbersome approach, which is a current limitation for routinely clinical use. Additionally, an important design consideration was the utilization of sub-atmospheric pressure alongside an opening in the liner at the area of interest. Although this liner opening might affect stump socket interactions, we expect that the relative movement of the residual bone was minimal, given that the liner maintained contact with the stump and socket around the liner’s opening. It is expected that it would be challenging to test alternative prosthetic fitting systems due to the movement of air within the socket during measurements, which needs to be explored in future research.

## Conclusion

This is the first study to successfully track in vivo residual bone movement through a prosthetic socket while executing different stepping tasks. The signal-to-noise ratios together with the consistencies of movement patterns within conditions shows that it is feasible to use B-mode US to visualize residual bone movement within a prosthetic socket during stepping tasks. Besides consistency of residual limb movement within subjects and tasks, differences in amplitudes between individuals and stepping directions were observed, ranging between 0.03 to 0.88 cm for medial–lateral movement and 0.14 to 0.87 cm for the anterior–posterior movement over all stepping tasks. The proposed method has the potential to enhance the understanding of the causes and effects of residual bone movement within the prosthetic socket.

### Supplementary Information

Supplementary Legends.

Supplementary Figure S2.

Supplementary Figure S3.

Supplementary Video S1.

## Data Availability

The data sets used and/or analysed during the current study are available from the corresponding author upon reasonable request.

## References

[1] List EB, Krijgh DD, Martin E, Coert JH (2021). Prevalence of residual limb pain and symptomatic neuromas after lower extremity amputation: A systematic review and meta-analysis. Pain.

[2] Bramley JL (2021). Changes in tissue composition and load response after transtibial amputation indicate biomechanical adaptation. Ann. Biomed. Eng..

[3] Powell JE, Sparling TL, Yuan X (2022). Diagnostic ultrasound of the residual limb: A narrative review. PM R.

[4] Frossard L, Conforto S, Aszmann OC (2022). Editorial: Bionics limb prostheses: Advances in clinical and prosthetic care. Front. Rehabil. Sci..

[5] Jan YK, Major MJ, Pu F, Sonenblum SE (2022). Editorial: Soft tissue biomechanics in wound healing and prevention. Front. Bioeng. Biotechnol..

[6] Graser M, Day S, Buis A (2020). Exploring the role of transtibial prosthetic use in deep tissue injury development: A scoping review. BMC Biomed. Eng..

[7] Frossard L (2023). Next-generation devices to diagnose residuum health of individuals suffering from limb loss: A narrative review of trends, opportunities, and challenges. J. Sci. Med. Sport.

[8] Ranger BJ (2019). 3D ultrasound imaging of residual limbs with camera-based motion compensation. IEEE Trans. Neural Syst. Rehabil. Eng..

[9] Davenport P, Noroozi S, Sewell P, Zahedi S (2017). Systematic review of studies examining transtibial prosthetic socket pressures with changes in device alignment. J. Med. Biol. Eng..

[10] Young PR, Hebert JS, Marasco PD, Carey JP, Schofield JS (2023). Advances in the measurement of prosthetic socket interface mechanics: A review of technology, techniques, and a 20-year update. Expert Rev. Med. Devices.

[11] Al-Fakih E, Abu Osman N, Mahmad Adikan F (2016). Techniques for interface stress measurements within prosthetic sockets of transtibial amputees: A review of the past 50 years of research. Sensors.

[12] Brienza DM, Campbell KE, Sprigle S (2022). The past, present, and future of pressure injury prevention in patients with spinal cord injury. Adv. Skin Wound Care.

[13] Mak AFT, Zhang M, Tam EWC (2010). Biomechanics of pressure ulcer in body tissues interacting with external forces during locomotion. Annu. Rev. Biomed. Eng..

[14] Grevsten S, Erikson U (1975). A roentgenological study of the stump-socket contact and skeletal displacement in the PTB-suction prosthesis. Upsala J. Med. Sci..

[15] Lilja M, Johansson T, Öberg T (1993). Movement of the tibial end in a PTB prosthesis socket: A sagittal X-ray study of the PTB prosthesis. Prosthet. Orthot. Int..

[16] Madsen MT, Haller J, Commean PK, Vannier MW (2000). A device for applying static loads prosthetic limbs of transtibial amputees during spiral examination. J. Rehabil. Res. Dev..

[17] Laprè, A. K., Nguyen, V. Q., Baspinar, U., White, M. & Sup, F. C. Capturing prosthetic socket fitment: Preliminary results using an ultrasound-based device. *IEEE Int. Conf. Rehabil. Robot.* 1221–1226. 10.1109/ICORR.2017.8009416 (2017). 10.1109/ICORR.2017.8009416 28813988

[18] Douglas T, Solomonidis S, Sandham W, Spence W (2002). Ultrasound imaging in lower limb prosthetics. IEEE Trans. Neural Syst. Rehabil. Eng..

[19] Papaioannou G, Mitrogiannis C, Nianios G, Fiedler G (2010). Assessment of amputee socket-stump-residual bone kinematics during strenuous activities using Dynamic Roentgen Stereogrammetric Analysis. J. Biomech..

[20] Klauser AS, Peetrons P (2010). Developments in musculoskeletal ultrasound and clinical applications. Skelet. Radiol..

[21] Ranger, B. J. *et al.* 3D optical imagery for motion compensation in a limb ultrasound system. 10.1117/12.2218386 (2016).

[22] Chong SY, Röhrle O (2016). Exploring the use of non-image-based ultrasound to detect the position of the residual femur within a stump. PLoS One.

[23] Klasen S, Uplegger C, Rensch S, Bächle T, Schneider U (2009). Ultrasound Pre-study Kinemat. Residual Tibia within a Trans-Tibial Socket Dur. Gait.

[24] Convery P, Murray KD (2000). Ultrasound study of the motion of the residual femur within a transfemoral socket during gait. Prosthet. Orthot. Int..

[25] Convery P, Murray KD (2001). Ultrasound study of the motion of the residual femur within a trans-femoral socket during daily living activities other than gait. Prosthet. Orthot. Int..

[26] WMA Declaration of Helsinki—Ethical Principles for Medical Research Involving Human Subjects—WMA—The World Medical Association. https://www.wma.net/policies-post/wma-declaration-of-helsinki-ethical-principles-for-medical-research-involving-human-subjects/19886379

[27] Pirouzi G (2014). Review of the socket design and interface pressure measurement for transtibial prosthesis. Sci. World J..

[28] Dickinson AS, Steer JW, Worsley PR (2017). Finite element analysis of the amputated lower limb: A systematic review and recommendations. Med. Eng. Phys..

