# Supplementary material for: Application of ultrasound to monitor in vivo residual bone movement within transtibial prosthetic sockets
Source: Sci Rep. 2024 Apr 27;14:9725. doi: 10.1038/s41598-024-60353-7 (PMC11055853; doi:10.1038/s41598-024-60353-7)
Supplement: Supplementary file 1 — Supplementary Legends. [file 41598_2024_60353_MOESM1_ESM.docx]

# Legends for supplementary information

**Video S1: Ultrasound video of residual bone movement while executing the backwards step condition**

This brief video demonstrates the movement of the residual limb during the execution of the backward task. The various layers depicted closely resemble those shown in Figure 3b.

**Figure S2: Mean time series for each condition compared within participants.**

Each color represents the mean curve of all data for the specific participant. All steps are normalized from 0 to 100% of the step and depicted on the x-axis, while the y-axis represents the absolute motion. For each step, the endpoint of the previous step was the starting point of the new step.

To note: the four metronome beats correspond approximately to the 0% mark (step initiation from baseline), 33% mark (step landing), 67% mark (returning step initiation), and 100% mark (baseline position).

**Figure S3: Measured anterior/posterior and medial lateral movement during “intact limb” and “in place” condition**

Each grey line corresponds to the residual bone movement during a single step in the prosthetic step conditions. The mean trajectory is depicted by the black line. The zero axis is positioned at the mean of the trajectory, which was utilized for the signal-to-noise ratio, representing the data most accurately. The three pictures at the top show the anterior/posterior motion and the bottom three the medial/lateral motion of the residual bone.

To note: the four metronome beats correspond approximately to the 0% mark (step initiation from baseline), 33% mark (step landing), 67% mark (returning step initiation), and 100% mark (baseline position).
