# Supplementary material for: Application of ultrasound to monitor in vivo residual bone movement within transtibial prosthetic sockets
Source: Sci Rep. 2024 Apr 27;14:9725. doi: 10.1038/s41598-024-60353-7 (PMC11055853; doi:10.1038/s41598-024-60353-7)
Supplement: Supplementary file 2 — Supplementary Figure S2. [file 41598_2024_60353_MOESM2_ESM.pdf]

Figure S2: Mean time series for each condition compared within participants.

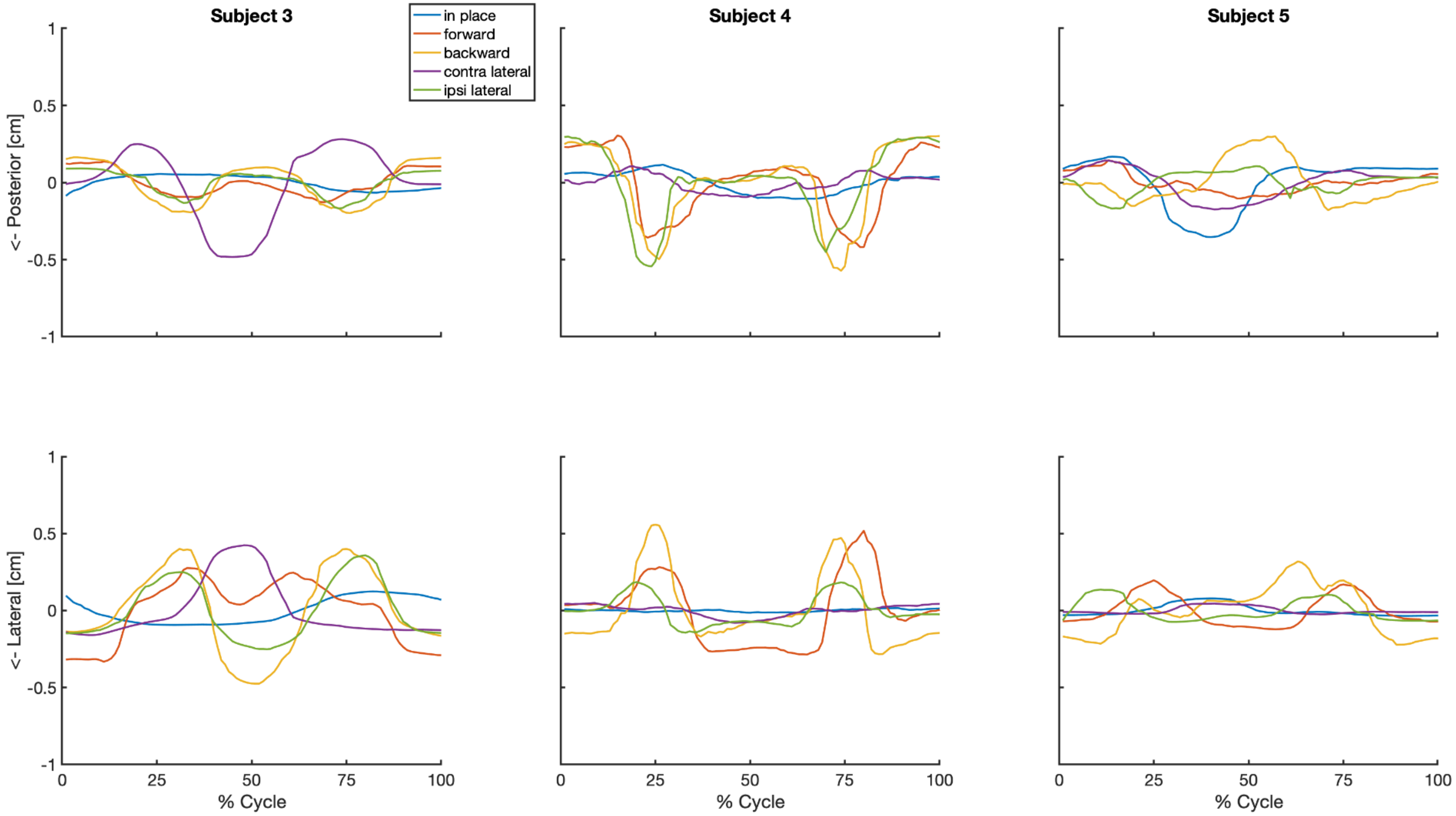

Each color represents the mean curve of all data for the specific participant. All steps are normalized from 0 to 100% of the step and depicted on the x-axis, while the y-axis represents the absolute motion. For each step, the endpoint of the previous step was the starting point of the new step.

To note: the four metronome beats correspond approximately to the 0% mark (step initiation from baseline), 33% mark (step landing), 67% mark (returning step initiation), and 100% mark (baseline position).
