# Supplementary material for: Application of ultrasound to monitor in vivo residual bone movement within transtibial prosthetic sockets
Source: Sci Rep. 2024 Apr 27;14:9725. doi: 10.1038/s41598-024-60353-7 (PMC11055853; doi:10.1038/s41598-024-60353-7)
Supplement: Supplementary file 3 — Supplementary Figure S3. [file 41598_2024_60353_MOESM3_ESM.pdf]

Figure S3: Measured anterior/posterior and medial lateral movement during “intact limb” and “in place” condition.

Sideways: Intact limb

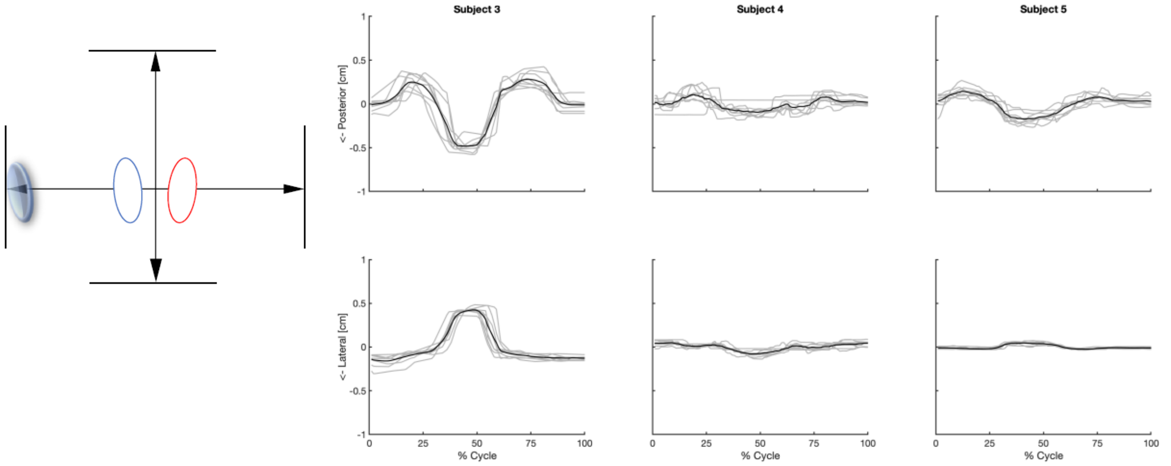

In place

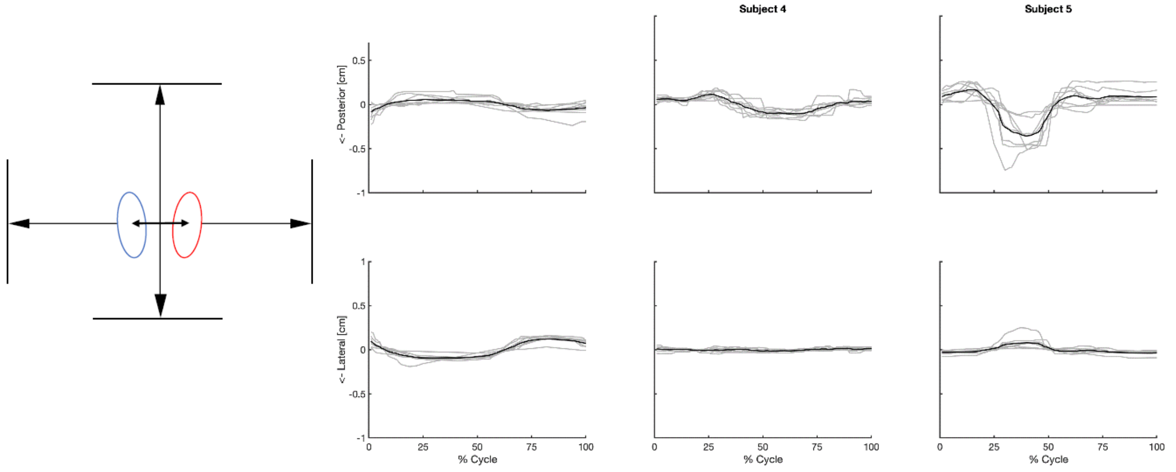

Each grey line corresponds to the residual bone movement during a single step in the prosthetic step conditions. The mean trajectory is depicted by the black line. The zero axis is positioned at the mean of the trajectory, which was utilized for the signal-to-noise ratio, representing the data most accurately. The three pictures at the top show the anterior/posterior motion and the bottom three the medial/lateral motion of the residual bone.

To note: the four metronome beats correspond approximately to the 0% mark (step initiation from baseline), 33% mark (step landing), 67% mark (returning step initiation), and 100% mark (baseline position).
